# Methods for assessing binding of a small-molecule radioligand to soluble enzyme isocitrate dehydrogenase 1 variants

**DOI:** 10.1186/s41181-026-00502-7

**Published:** 2026-09-22

**Authors:** Winnie Deuther-Conrad, Thu Hang Lai, Andreas Maurer, Barbara Wenzel

**Affiliations:** 1 Department of Experimental Neurooncological Radiopharmacy, Institute of Radiopharmaceutical Cancer Research, Research Site Leipzig, Helmholtz-Zentrum Dresden-Rossendorf (HZDR), 04318 Leipzig, Germany; 2 Department of Research and Development, ROTOP Pharmaka GmbH, 01328 Dresden, Germany; 3 Institute for Drug Discovery, Faculty of Medicine, Leipzig University, 04103 Leipzig, Germany

## Abstract

**Background:**

Isocitrate dehydrogenase 1 (IDH1) is a cytosolic NADP^+^-dependent enzyme involved in cellular redox homeostasis and 2-oxoglutarate (2-OG) metabolism. Glioma-associated mutations in IDH1 confer a neomorphic enzymatic activity, resulting in the production and accumulation of the oncometabolite D-2-hydroxyglutarate (2-HG), which contributes to metabolic and epigenetic alterations in IDH-mutant tumors. Small-molecule inhibitors of mutant IDH1 have emerged as promising therapeutic agents. In parallel, radiolabeled derivatives of these inhibitors are being investigated as potential positron emission tomography (PET) tracers for non-invasive imaging of mutant IDH1. Characterizing the binding of small-molecule radioligands to soluble intracellular proteins such as IDH1, however, presents specific methodological challenges. Appropriate experimental approaches are therefore needed to characterize radioligand binding to IDH1 enzymes.

**Results:**

Using several established biochemical and biophysical approaches, we found that the radiofluorinated inhibitor ivosidenib ([¹⁸F]AG-120) binds specifically to both IDH1 and IDH1^R132H^ at nanomolar radioligand concentrations. However, the investigated approaches yielded method-dependent apparent affinity estimates.

**Conclusions:**

The findings support the use of radioligand-based approaches for characterizing soluble intracellular protein targets, while highlighting the need for assay-specific optimization and careful interpretation of quantitative binding parameters.

**Supplementary Information:**

The online version contains supplementary material available at https://doi.org/10.1186/s41181-026-00502-7.

## Introduction

Isocitrate dehydrogenases (IDHs) catalyze the oxidative decarboxylation of isocitrate to 2-oxoglutarate (2-OG), coupled to the reduction of NADP⁺ or NAD⁺ to NADPH or NADH, thereby linking central metabolism to cellular redox homeostasis. Mammalian cells express three IDH isoenzymes: cytosolic, NADP⁺-dependent IDH1; mitochondrial, NADP⁺-dependent IDH2; and mitochondrial, NAD⁺-dependent IDH3. Mutations in IDH1 or IDH2 are present in approximately 70–80% of adult WHO grade 2 and 3 diffuse gliomas, with the heterozygous R132H substitution in IDH1 representing the predominant variant (Louis et al. 2021, Schaff et al. 2024, Yan et al. 2009, Parsons et al. 2008). The R132H mutation confers neomorphic enzymatic activity, resulting in NADPH-dependent reduction of 2-OG to the oncometabolite 2-HG (Dang et al. 2009). Consequently, 2-HG accumulates to high intracellular concentrations in IDH-mutant gliomas and competitively inhibits multiple 2-OG-dependent dioxygenases involved in epigenetic regulation and cell differentiation (Xu et al. 2011). Despite these tumor-promoting effects, IDH-mutant gliomas generally exhibit a more favorable prognosis, reflecting their distinct tumor biology and greater sensitivity to chemo- and radiotherapy (Pirozzi and Yan 2021). The high intracellular concentrations of 2-HG in IDH-mutant gliomas also provide a basis for non-invasive assessment of IDH mutation status by ^1^H-MR spectroscopy (McHugh et al. 2025). However, definitive determination of IDH status currently relies on molecular characterization of tumor tissue, which is essential for accurate diagnosis and treatment stratification (Weller et al. 2021). IDH-mutant gliomas generally exhibit a more favorable clinical course than IDH-wildtype gliomas, and IDH status constitutes a defining molecular feature of distinct adult-type diffuse glioma entities in the current WHO classification (Louis et al. 2021). Importantly, IDH status influences treatment strategies through molecular classification and has become therapeutically actionable (Blakeley et al. 2025). This has motivated the development of small-molecule inhibitors targeting mutant IDH1 (mIDH1), which predominantly bind allosterically at the enzyme dimer interface and suppress the neomorphic production of 2-HG, thereby targeting the metabolic and epigenetic consequences of the mutation. Several mIDH inhibitors have entered clinical development, including ivosidenib (AG-120) (Popovici-Muller et al. 2018), the first mIDH1-selective small molecule approved for cancer therapy (Dhillon 2018).

The clinical relevance of IDH mutation status has motivated efforts to develop non-invasive imaging approaches for its assessment in vivo. PET radioligands targeting mIDH1 may enable direct visualization of the target protein and provide spatial information on IDH1-mutant tumor tissue. Accordingly, several PET-radioligand development efforts have been reported using structurally different low-molecular weight mIDH1 inhibitors for the development of PET radioligands (Neumaier et al. 2023), with a radiofluorinated analogue of AG-120 synthesized in our laboratories and recently evaluated in vitro and in vivo (Lai et al. 2024). In our previous study, [^18^F]AG-120 showed approximately 30-fold higher uptake in IDH1^R132H^-overexpressing U251 glioblastoma cells (IDH1^R132H^-U251) than in wild-type IDH1 transfected cells (IDH1^wt^-U251), whereas PET imaging of rats bearing glioblastomas generated from the same cell lines revealed only moderately higher accumulation in IDH1^R132H^ tumors. This raised the question of whether differences in the interaction of [^18^F]AG-120 with the two IDH1 variants in the cellular system used in our previous study might contribute to this. In this context, recent biophysical and biochemical studies using label-free approaches have shown that the functional selectivity of allosteric mIDH inhibitors does not necessarily reflect preferential binding to the mutant enzyme. Instead, selectivity may result from differential effects on metal ion and substrate binding in IDH1^R132H^ and IDH1^wt^(Deng et al. 2015, Liu et al. 2021, 2023). These studies, including investigations of AG-120, reported comparable binding affinities for both isoforms in the high-nanomolar range. Whether this binding profile is also observed for [^18^F]AG-120 at tracer concentrations in the cellular system used in our previous uptake studies had not been established.

**Scheme 1:**
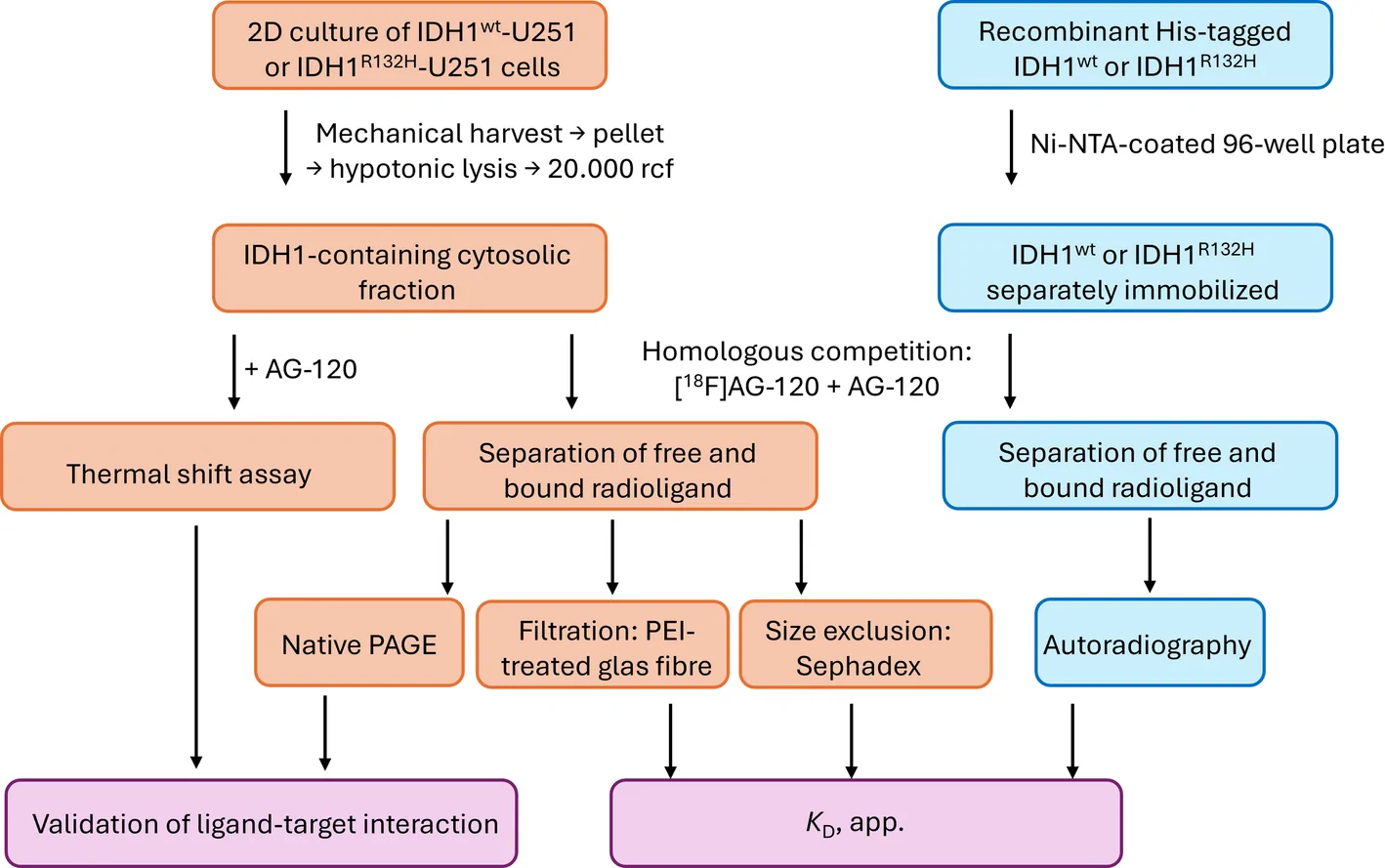
The general study design and workflow of the binding experiments. Abbreviations: 2D = 2-dimensional; rcf = relative centrifugal force; IDH1 = isocitrate dehydrogenase 1; Ni-NTA = nickel-nitrilotriacetic acid; PAGE = polyacrylamide gel electrophoresis; PEI = polyethylenimine

The aim of this study was not to establish a universally applicable protocol for determining ligand affinity or specificity for IDH1^R132H^, but to evaluate a set of established biochemical and biophysical approaches for radioligand binding studies using the resources available in our laboratory. Using standard radiopharmaceutical laboratory equipment, we characterized the in vitro binding of [¹⁸F]AG-120 to IDH1^wt^ and IDH1^R132H^ and estimated apparent binding affinities. As outlined in Scheme 1, the experiments were performed using cytosolic fractions from stably transfected U251 cells overexpressing either IDH1^wt^ or IDH1^R132H^ as well as recombinant His-tagged IDH1^wt^ and IDH1^R132H^ immobilized via metal-chelate binding. Radioligand binding was assessed by size-exclusion chromatography, filtration, and autoradiography, while thermal shift assays and Western blotting provided additional evidence for target engagement and target identity, respectively. The high sensitivity of radioactivity detection enabled binding to be assessed at low radioligand concentrations.

## Results

### Analysis of the interaction of AG-120 with IDH1 in transfected IDH1^wt^-U251 and IDH1^R132H^-U251 cells

**Fig. 1:**
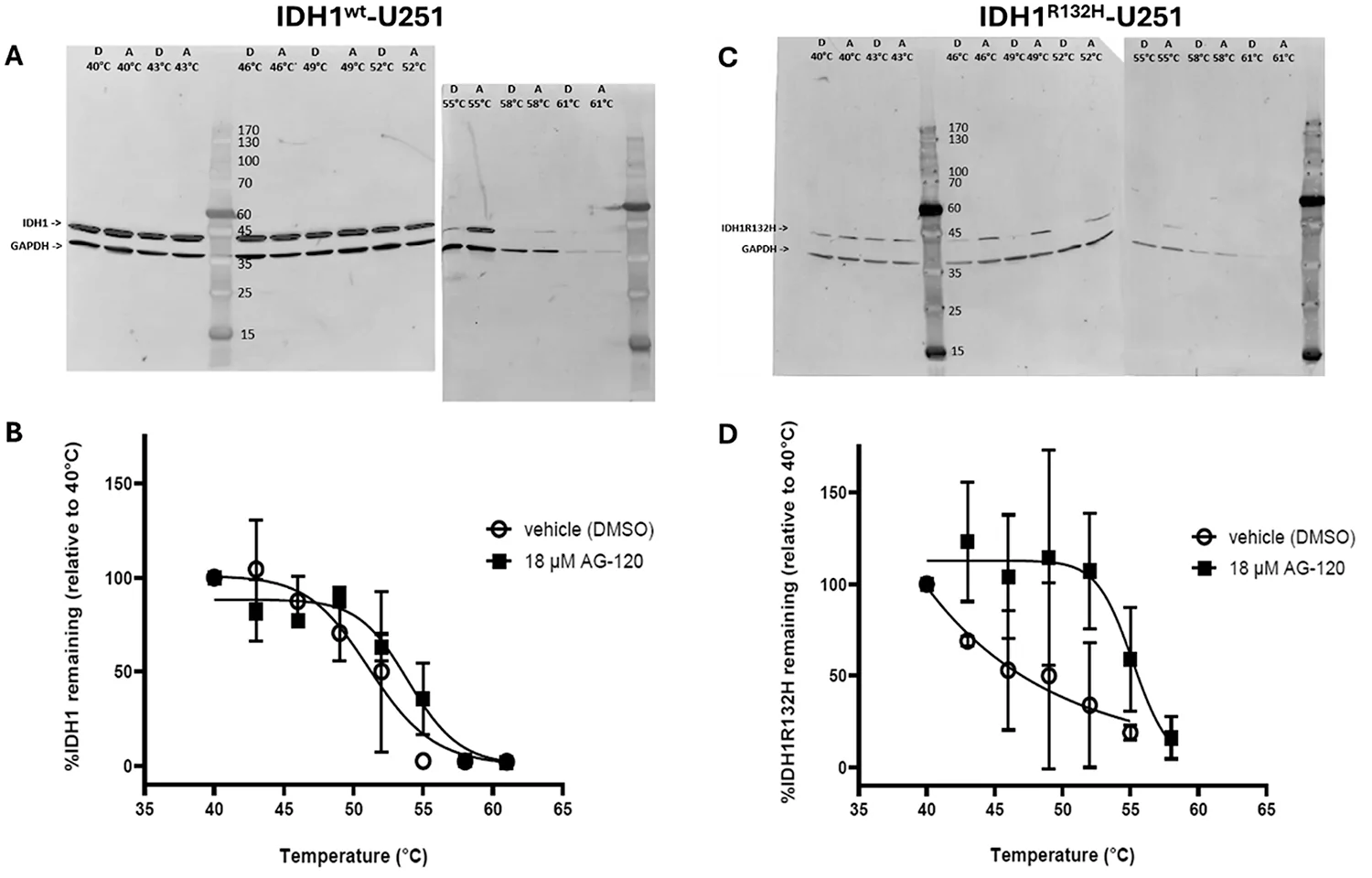
Thermal Shift Assay. Effect of AG-120 on the thermal stability of IDH1^wt^ (**A**) and IDH1^R132H^ (**C**), assessed by Western blotting using a pan-IDH1 or IDH1R132H-specific antibody, respectively. Representative experiments are shown. Abbreviations: D = DMSO (vehicle control); A = 18 µM AG-120. (**B**, **D**) Densitometric analysis of immunoblots shown in (**A**) and (**C**), respectively. Band intensities corresponding to IDH1^wt^ or IDH1^R132H^ were quantified by densitometry and expressed as percentages relative to the intensity measured at 40 °C. The resulting values were plotted against temperature and fitted using a four-parameter sigmoidal model. The midpoint of each curve, representing the temperature at which 50% of the initial band intensity remained, was defined as the apparent aggregation temperature (T_agg_). T_agg_ values for the representative experiments are shown as follows: IDH1^wt^, vehicle = 48.9 °C and 18 µM AG-120 = 53.3 °C; IDH1^R132H^: vehicle = 48.6 °C and 18 µM AG-120 = 55.1 °C

To investigate target engagement of AG-120 in the cytosolic fractions of the transfected cell lines used for our in vitro and in vivo studies, thermal shift-Western blot assays were performed. Cytosolic fractions from IDH1^wt^-U251 and IDH1^R132H^-U251 cells were incubated with either DMSO (0.18% working concentration; vehicle control) or 18 µM AG-120 for 60 min at room temperature. Aliquots were subsequently incubated at different temperatures (40 –67 °C) for 3 min, and the soluble fractions were analyzed by Western blotting using either a pan-IDH1 antibody or an IDH1^R132H^-specific antibody. As shown in Fig. 1, AG-120 increased the thermal stability of both IDH1^wt^ and IDH1^R132H^ compared with vehicle-treated controls, providing evidence of target engagement in the respective cytosolic fractions. Quantitative densitometric analysis of the Western blot bands (Fig. 1A, C) revealed an increase in the apparent aggregation temperature (T_agg_), defined as the temperature at which 50% of the initial soluble protein signal was lost (Fig. 1B, D). The T_agg_ values determined for the wild-type and mutant enzymes (50.5 ± 2.0 °C, *n* = 7, and 45.6 ± 1.5 °C, *n* = 8, respectively) were comparable to those reported for the corresponding purified homodimeric enzymes using fluorescence-based thermal shift analysis (53.0 °C ad 47.2 °C, respectively), both in terms of their absolute values and the difference between the two variants (Pietrak et al. 2011). At 18 µM AG-120, increases in T_agg_ (ΔT_agg_) of 3.6 °C for IDH1^wt^ and 9.8 °C for IDH1^R132H^ were observed, corresponding to mean values from two independent experiments (IDH1^wt^: 4.3 °C and 2.8 °C; IDH1^R132H^: 9.5 °C and 10.0 °C). This is consistent with target engagement in the cytosol-enriched preparations from transfected U251 cells.

These results indicated that (i) cytosolic fractions from IDH1^wt^-U251 and IDH1^R132H^-U251 cells are suitable for investigating the interaction of AG-120 with the respective IDH1 variants, and (ii) AG-120 stabilizes both IDH1 variants in cytosolic thermal shift assays, providing evidence for target engagement.

**Fig. 2:**
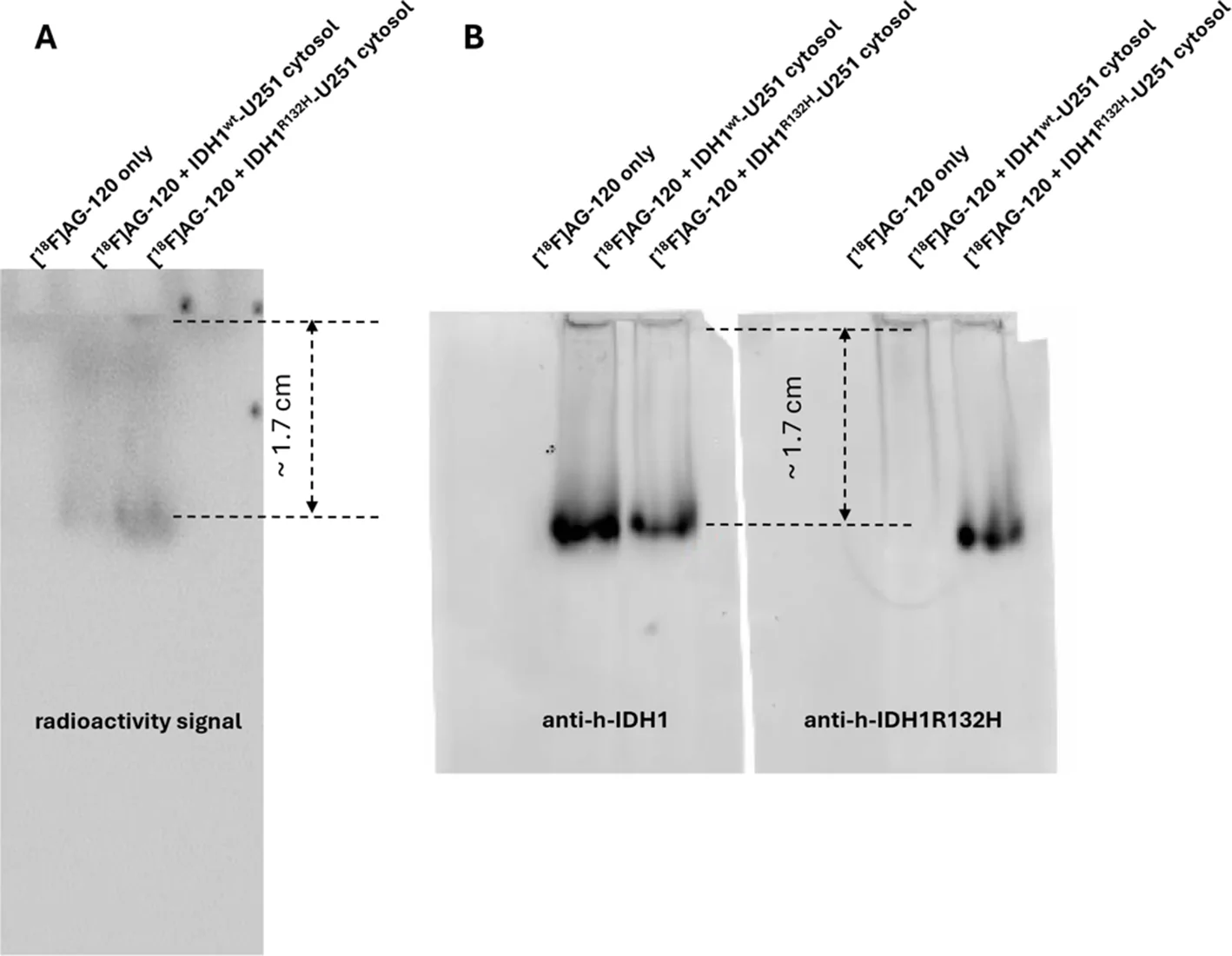
PAGE autoradiography and Western blot. Cytosolic fractions from IDH1^wt^-U251 or IDH1^R132H^-U251 cells were separated by native polyacrylamide gel electrophoresis 90 min after incubation with [^18^F]AG-120, followed by autoradiographic analysis of the gel. Subsequently, proteins were transferred to a PVDF membrane and the respective enzymes were detected by fluorescence immunostaining (3 independent experiments). (**A**) Autoradiography of a representative experiment (working concentration [^18^F]AG-120 = 1 nM; protein concentration: IDH1^wt^-U251 cytosol = 3.5 mg/mL, IDH1^R132H^-U251 cytosol = 3.4 mg/mL). (**B**) Corresponding Western blot (anti-h-IDH1 = Dianova W-09, 1:1000; anti-h-IDH1R132H = Dianova H-09, 1:1000)

To further investigate whether [^18^F]AG-120 binding in the cytosolic fractions could be attributed to the respective IDH1 variant, we performed radioligand binding experiments using nanomolar concentrations of [^18^F]AG-120. Following incubation, the samples were separated by native PAGE and analyzed by autoradiography and Western blotting after transfer to a PVDF membrane, using either a pan-IDH1 antibody or an IDH1^R132H^-specific antibody. In a representative experiment (Fig. 2), the radioactive signal co-localized with the corresponding IDH1^wt^ or IDH1^R132H^ band detected by Western blotting, supporting an association of [^18^F]AG-120 with the respective IDH1 variant. However, the contribution of co-migrating proteins cannot be excluded. Persistence of the radioactive signal during sample preparation and native PAGE is consistent with a sufficiently stable association under these experimental conditions. Additional radioactive signals observed in the cytosolic fractions, but absent in the pure radioligand sample, were detected in regions corresponding to more slowly migrating protein species than the enzyme of interest. These signals may reflect nonspecific binding of the radioligand to cytosolic proteins other than the target enzyme.

Together with the thermal shift data indicating that AG-120 stabilizes both IDH1^wt^ and IDH1^R132H^ at micromolar concentrations (Fig. 1), the co-migration of the [^18^F]AG-120-derived radioactive signal with the respective IDH1 band supports target engagement by the radioligand. However, co-migration after native PAGE does not exclude binding to an off-target protein or protein complex with similar electrophoretic mobility. Thus, the results shown in Fig. 2 provide supportive, but not definitive evidence for IDH1 as the binding target.

Based on these findings, cytosolic fractions from IDH1^wt^-U251 and IDH1^R132H^-U251 cells were subsequently used for quantitative radioligand binding experiments to characterize [^18^F]AG-120 binding and estimate apparent affinities.

### Use of the transfected cells for the evaluation of the binding specificity and affinity of [^18^F]AG-120 to IDH1^wt^ and IDH1^R132H^

To further investigate the binding affinity and specificity of AG-120, homologous radioligand binding studies were performed using the cytosolic fractions of IDH1^wt^- or IDH1^R132H^-transfected U251 cells. A fixed concentration of [^18^F]AG-120 was competed with increasing concentrations of unlabeled AG-120 to determine the apparent binding affinity. Binding was assessed by size-exclusion chromatography (SEC) or by rapid glass fiber filtration, using different approaches to separate free from protein-bound radioligand.

**Fig. 3:**
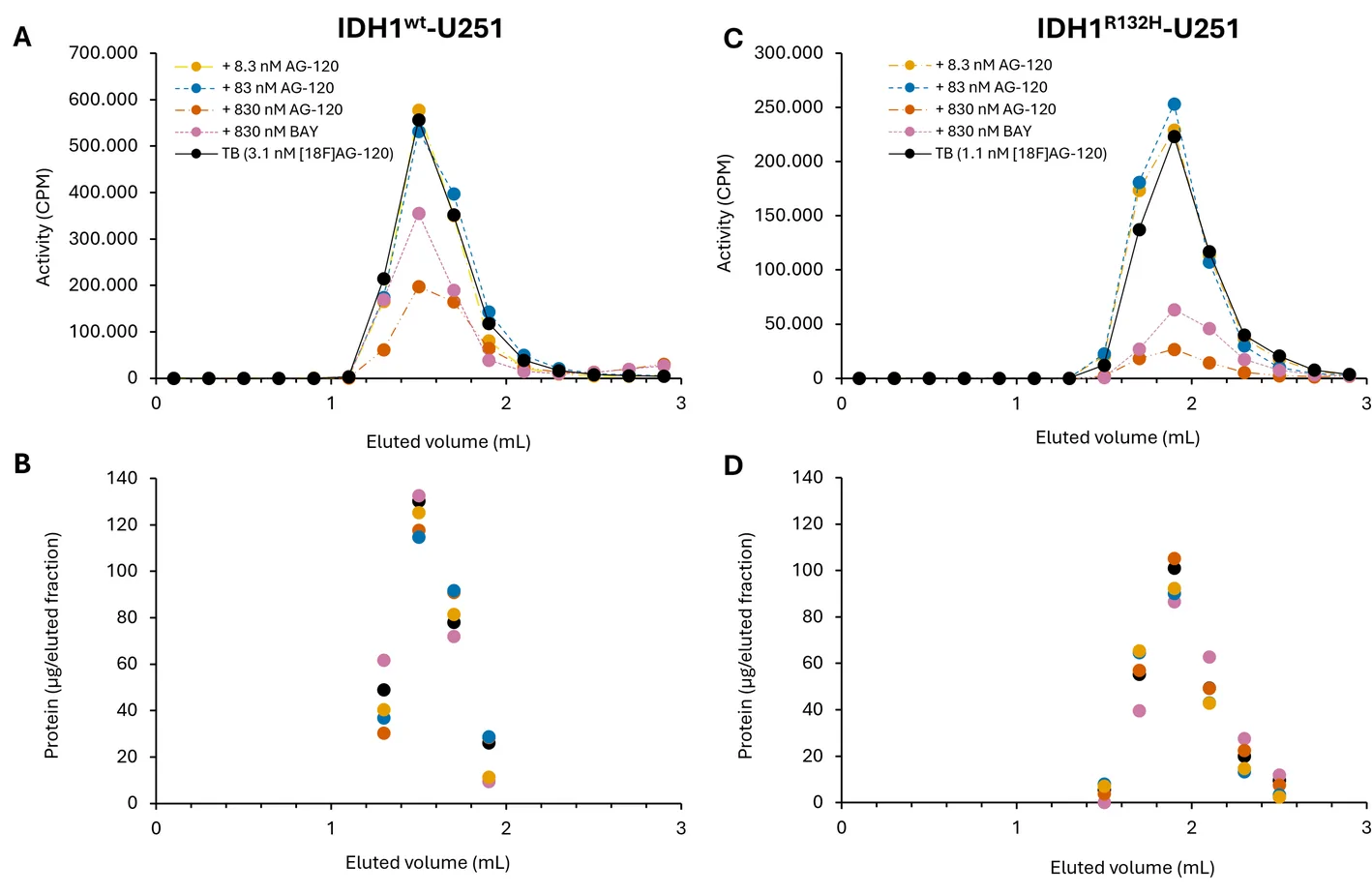
Size-exclusion chromatography of [^18^F]AG-120 binding in cytosolic fractions from transfected U251 cells. Elution profiles of radioactivity (**A**, **C**) and protein concentration (**B**, **D**) from cytosolic fractions of IDH1^wt^-U251 (**A**, **B**) and IDH1^R132H^-U251 (**C**, **D**) cells, respectively, following size-exclusion chromatography on Sephadex G-25. Radioactivity is shown as counts per minute (CPM) per eluted volume, and protein concentration as µg per eluted volume. For homologous competition experiments, cytosolic fractions were incubated with a fixed concentration of [^18^F]AG-120 alone (total binding, TB) or with increasing concentrations of AG-120, as indicated. Nonspecific binding was assessed by co-incubation with BAY1436032 (BAY). Representative elution profiles from one independent experiment performed with IDH1^wt^ and one performed with IDH1^R132H^ are shown. Two independent experiments were performed for each enzyme

Initially, SEC as a low-throughput method for studying receptor–ligand interactions (Hummel and Dreyer 1962, Winzor 2000) was used to separate protein-bound [^18^F]AG-120 from free radioligand. Cytosolic fractions from IDH1^wt^- or IDH1^R132H^-transfected U251 cells were pre-incubated with nanomolar concentrations of [^18^F]AG-120 alone (total binding), together with the pan-IDH1 inhibitor BAY1436032 at 830 nM (non-specific binding), or with increasing concentrations of unlabeled AG-120 (nM–µM) for homologous competition. Samples were subsequently separated by stepwise elution on Sephadex G-25 (exclusion limit ~ 5 kDa). The radioligand co-eluted with protein in the earliest eluting fractions, as indicated by overlapping radioactivity and protein elution profiles (Fig. 3). Co-incubation with either BAY1436032 or increasing concentrations of AG-120 reduced the radioactivity-associated peak, as reflected by a decrease in its area under the curve (AUC) (Fig. 3A, C). IDH1 was detected in the corresponding protein-containing fractions (Fig. 3B, D) by subsequent Western blotting (Supplement: Fig. S1). A control experiment comparing radioligand elution in the presence and absence of protein is shown in Supplementary Figure S2. In the absence of protein, most of the radioligand remained on the column and did not co-elute with the protein-containing fractions.

However, inhibition curves could not be reliably generated from the AUC values, as substantial inhibition of [^18^F]AG-120 binding occurred only at concentrations of either BAY1436032 or AG-120 approximately 1000-fold higher than those used for the radioligand (Fig. 3A, C). For the homologous competition experiments with AG-120, concentration-dependent inhibition was therefore converted into estimates of specific binding to derive apparent saturation curves (Supplement: Figure S3, S4). This analysis yielded apparent *K*_D_ values of 541 nM and 40 nM for binding of [^18^F]AG-120 to cytosolic fractions of IDH1^wt^- and IDH1^R132H^-U251 cells, respectively, representing mean values from two independent experiments for each enzyme (IDH1^wt^: 465 and 617 nM; IDH1^R132H^: 37 and 42 nM), although B_max_ values had to be fixed during fitting. Inspection of the underlying data, however, indicated substantial ligand depletion, with more than 10% of the total applied radioligand retained in the protein-bound fraction. In total binding samples, this fraction was > 95% for IDH1^wt^-U251 and ~ 70% for IDH1^R132H^-U251, while in the corresponding nonspecific samples it was ~ 70% and ~ 20%, respectively. Thus, the free radioligand concentration could not be assumed to equal the total radioligand concentration. The data suggest a greater proportion of competitor-displaceable binding in cytosolic fractions from IDH1^R132H^- compared to IDH1^wt^-U251 cells (~ 80% vs. ~30% of total binding, respectively), but the extent of ligand depletion precluded reliable estimation of binding parameters from the SEC experiments.

**Fig. 4:**
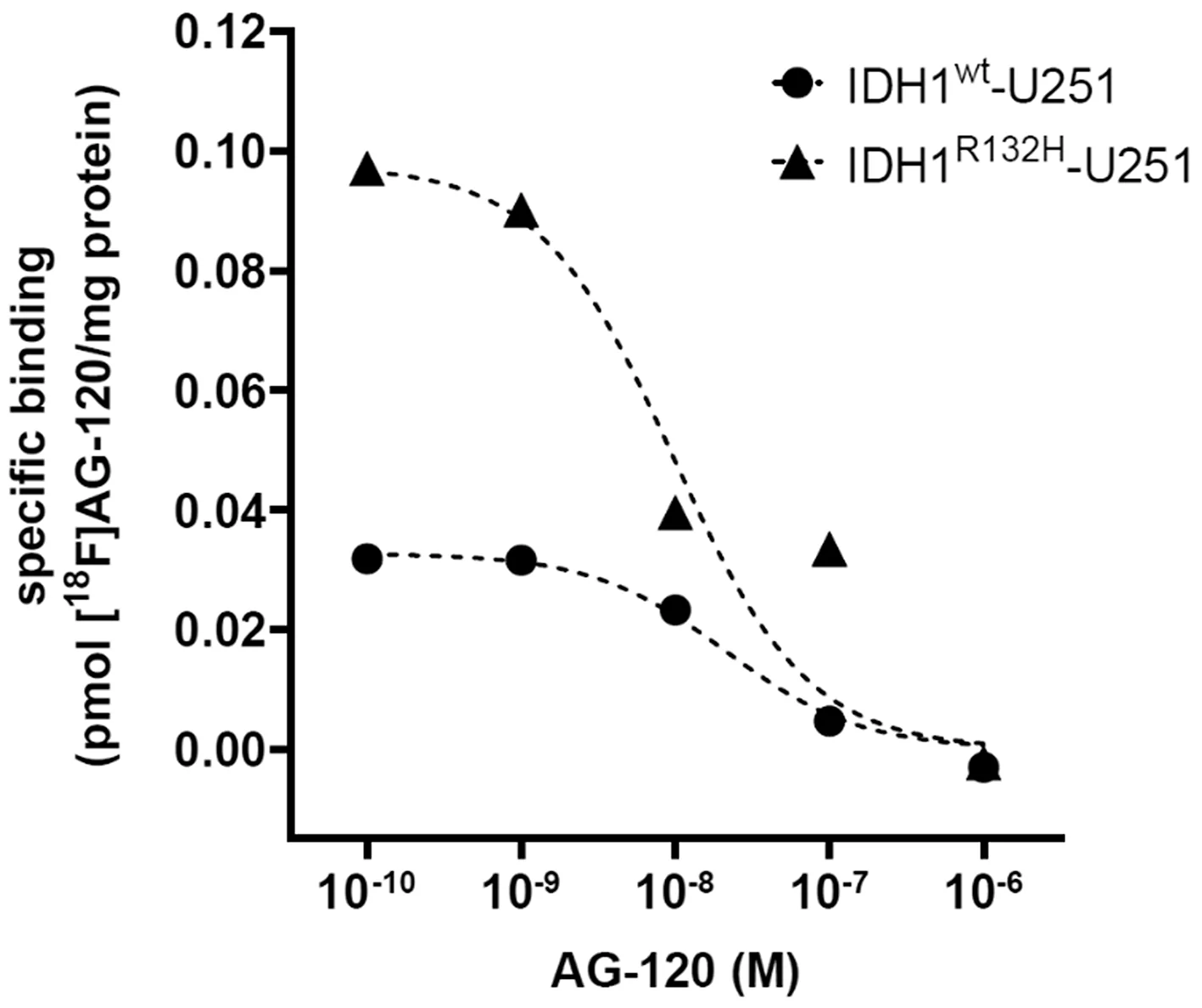
Representative inhibition curves from a homologous competition experiment using cytosolic fractions of IDH1^wt^-U251 and IDH1^R132H^-U251 cells. Protein-bound and free [^18^F]AG-120 were separated by glass fiber filtration. The final concentration of [^18^F]AG-120 was 1.9 nM. Nonspecific binding was determined by co-incubation with 1 µM BAY1436032. Final protein concentrations were 194 µg/mL and 191 µg/mL for IDH1^wt^-U251 and IDH1^R132H^-U251 samples, respectively. IC_50_ values were 21.3 nM for IDH1^wt^-U251 and 9.71 nM for IDH1^R132H^-U251

To reduce ligand depletion, homologous competition experiments were subsequently performed using lower protein concentrations and rapid filtration to separate protein-bound from free radioligand. Using polyethyleneimine (PEI), a strongly cationic polymer at pH 7.4, soluble proteins can be retained by filtration on preincubated glass fiber filters (Bruns et al. 1983). Under these conditions, the fraction of [^18^F]AG-120 bound to cytosolic proteins remained below 10% of the total applied radioligand. Nonspecific binding, determined by co-incubation with 1 µM BAY1436032, accounted for approximately 60% and 35% of total binding in cytosolic fractions from IDH1^wt^- or IDH1^R132H^-transfected U251 cells, respectively. Homologous inhibition curves derived from these experiments (*n* = 2 independent experiments for each enzyme; representative experiment shown in Fig. 4) yielded apparent *K*_D_ values of 19.9 nM for IDH1^wt^-U251 and 9.37 nM for IDH1^R132H^-U251 cells.

As a control, a glass fiber filtration binding assay was performed using cytosolic fraction prepared from parental U251 cells expressing endogenous IDH1 at physiological level. The protein-bound fraction of [^18^F]AG-120 was not measurably reduced by 1 µM BAY1436032 or AG-120 compared with control, indicating no detectable inhibition under the assay conditions (Supplement, Figure S5). However, low-level specific binding to endogenous IDH1 cannot be excluded.

In summary, [^18^F]AG-120 bound to both IDH1 variants in cytosolic preparations from IDH1^wt^-U251 and IDH1^R132H^-U251 cells, which express the respective enzymes at high levels (Kessler et al. 2015). Binding was observed at submicromolar concentrations. Across the investigated assay formats, the apparent *K*_D_ estimates were consistently lower for IDH1^R132H^ than for IDH1^wt^ although the limited number of independent experiments precludes a robust comparison between the two variants. The fraction of specific binding was also higher in cytosolic preparations from IDH1^R132H^-U251 cells.

### Use of recombinant His-tagged IDH1 enzymes for the evaluation of the binding specificity and affinity of [^18^F]AG-120

**Fig. 5:**
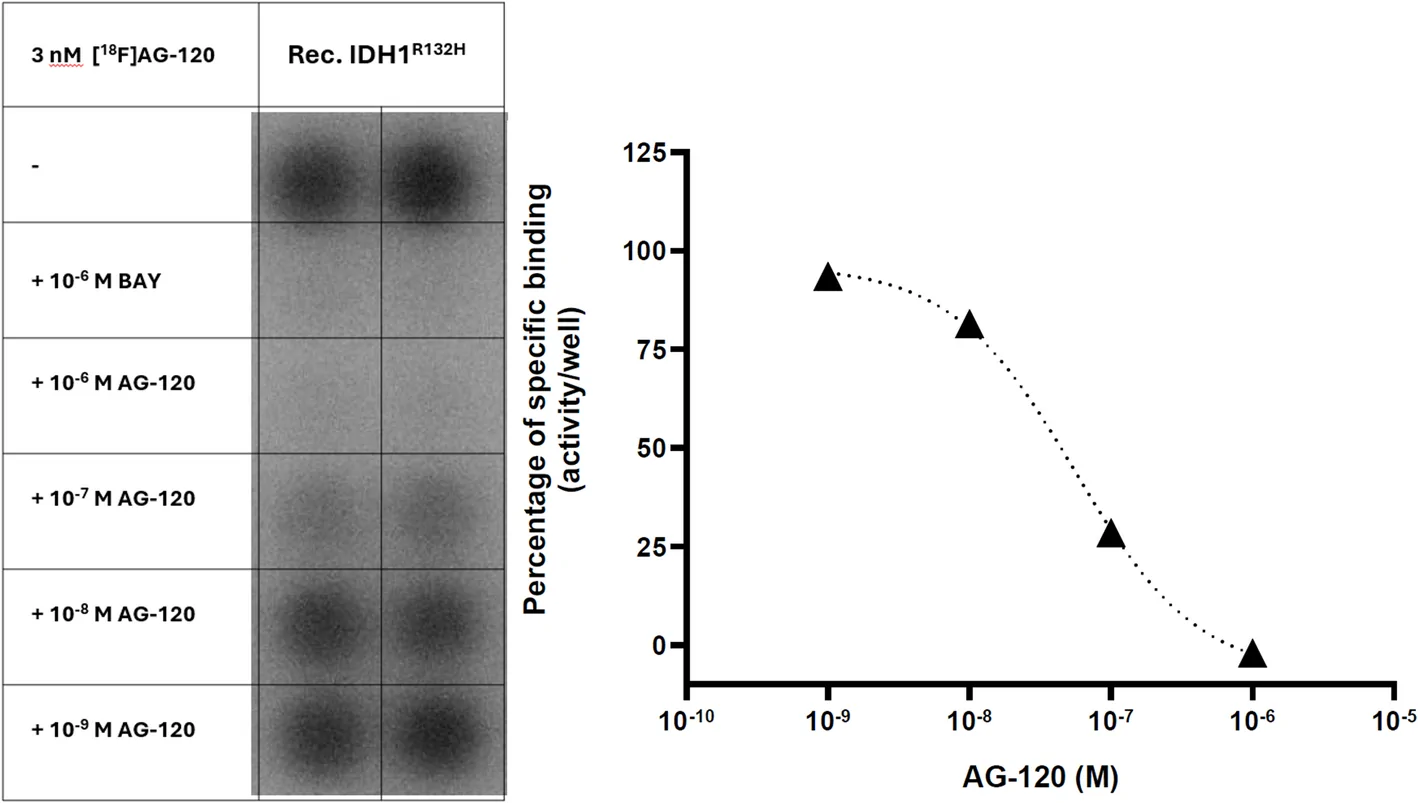
Determination of the affinity of [^18^F]AG-120 using immobilized His-tagged recombinant IDH1 enzymes. Representative experiment performed by incubation of His-tagged IDH1^R132H^ immobilized on a Ni-NTA HisSorb plate (384 ng protein/well) with 3.3 nM [^18^F]AG-120 alone (total binding) or in the presence of 1 µM BAY1436032 (nonspecific binding) or increasing concentrations of AG-120 (1 nM – 1 µM). Autoradiographic image obtained by phosphorimaging of the well plate. Inhibition curve generated by nonlinear regression analysis of data derived from densitometric analysis of the autoradiographic image, plotted as specific binding of [^18^F]AG-120 vs. the concentration of AG-120 (apparent K_D_ = 52.2 nM)

To simplify the binding assay system and reduce the complexity of cytosolic preparations, purified recombinant His-tagged IDH1 and IDH1^R132H^ were subsequently used for binding experiments with nanomolar concentrations of [^18^F]AG-120. The enzymes were immobilized on Ni-coated well plates, and bound and free radioligand were separated by washing. Apparent K_D_ values were determined from homologous competition experiments using increasing concentrations of AG-120, with nonspecific binding determined by co-incubation with 1 µM BAY1436032. Bound radioligand was quantified by densitometric analysis of phosphorimaging autoradiographs of the well plates. The fraction of bound radioligand remained below 10% of the total applied dose. For IDH1^R132H^, apparent K_D_ values ranged from approximately 50–200 nM (101 ± 68.4 nM; *n* = 4). A representative experiment is shown in Fig. 5.

However, apparent *K*_D_ values varied between batches of recombinant His-tagged IDH1^R132H^. Two independent experiments were performed using two different batches of the enzyme, yielding apparent *K*_D_ values of approximately 50 nM (52.2 and 51.8 nM) and 150 nM (104 and 197 nM), respectively. Given the limited number of measurements, no firm conclusions regarding batch-dependent variability can be drawn.

Although equal amounts of His-tagged IDH1 and His-tagged IDH1^R132H^, based on the manufacturer’s protein concentration specifications, were used for preparation of the Ni-coated well plates, the actual amount of protein immobilized on the wells could not be quantified by BCA assay due to the BSA-preblocked plate format. Autoradiography consistently showed substantially lower [^18^F]AG-120-associated signal for IDH1 than for IDH1^R132H^. Consequently, only a single inhibition experiment for His-tagged IDH1 could be evaluated, yielding an apparent K_D_ value of 182 nM.

The experiments with recombinant His-tagged enzymes were broadly consistent with the findings obtained using cytosolic fractions from the transfected cells. Apparent [^18^F]AG-120 binding was detected for both enzymes, with higher radioligand-associated signal observed for IDH1R132H than for IDH1; however, no statistically robust comparison between variants was possible. Differences in immobilization efficiency related to the different His-tag positions (N-terminal in recombinant IDH1^wt^ and C-terminal in recombinant IDH1^R132H^) may have contributed to the observed signal difference. Mutation-induced structural changes affecting accessibility of the allosteric binding pocket (Yang et al. 2010) represent a possible additional explanation, but this was not investigated in the present study.

## Discussion

The study was motivated by our efforts to develop a radiofluorinated analogue of AG-120, a selective inhibitor of mutant IDH1^R132H^, and by the discrepancy observed in our previous work between the substantially higher cellular uptake of [^18^F]AG-120 in IDH1^R132H^-expressing cells compared with IDH1^wt^ cells in vitro and the absence of a corresponding difference in tumor accumulation in vivo (Lai et al. 2024). Previous biophysical studies investigating the mechanism of inhibition have shown that AG-120 and other allosteric IDH1 inhibitors bind to both wild-type and mutant IDH1, despite their preferential inhibitory activity toward the mutant enzyme at nanomolar concentrations (Liu et al. 2021, Liu et al. 2023). In the present study, binding of [^18^F]AG-120 to both IDH1^wt^ and IDH1^R132H^ was detected at low-nanomolar radioligand concentrations using recombinant proteins and cytosolic fractions derived from engineered cells (Kessler et al. 2015, Kessler et al. 2019). Competition with BAY1436032 further supported the specificity of the interaction. Submicromolar apparent affinities were obtained across three different assay formats, with lower apparent *K*_D_ values for IDH1^R132H^ than for IDH1^wt^. However, the apparent *K*_D_ values varied by approximately 2- to 30-fold between the assay formats, and the magnitude of the difference between IDH1^wt^ and IDH1^R132H^ varied substantially across methods (Table 1), indicating that the measured binding parameters are strongly influenced by assay conditions.

**Table 1:** Summary of apparent *K*_D_ values for AG-120 binding to IDH1 and IDH1^R132H^ determined by homologous competition assays with [^18^F]AG-120

Method	Source of enzyme	Technical replicates	Apparent *K*_D_	
			IDH1^wt^	IDH1^R132H^
Size-exclusion chromatography	Cytosolic fraction of transfected U251 cells	*n* = 1	541 nM (465 nM, 617 nM)	40 nM (37 nM, 42 nM)
Filtration	Cytosolic fraction of transfected U251 cells	*n* = 3	19.9 nM (18.5 nM, 21.3 nM)	8.37 nM (9.71 nM, 7.02 nM)
Ni-His plate autoradiography	His-tagged recombinant enzyme	*n* = 2	182 nM	101 ± 68.4 nM

Nonspecific binding was defined by co-incubation with BAY1436032, and different methods were used to separate bound from free radioligand. Values are reported as means of independent experiments; individual values are given in parentheses for approaches based on two independent experiments. For the Ni-His plate assay, the IDH1^wt^ estimate is based on one independent experiment, whereas the IDH1^R132H^ value represents four independent experiments (two with each of two protein batches), reported as mean ± SD. The numbers of technical replicates are indicated for each assay

The different assay formats represent distinct experimental conditions that can affect quantitative binding measurements. Size-exclusion chromatography involves relatively high protein concentrations and prolonged separation, whereas filtration permits rapid separation and lower protein concentrations; the Ni-His plate assay uses immobilized recombinant protein. Consequently, ligand depletion, immobilization-related effects, and non-equilibrium processes may contribute to the observed differences in apparent affinity. The filtration assay provided the lowest ligand depletion with the bound fraction remaining below 10%, and therefore gave the most robust quantitative estimate among the approaches investigated. Nevertheless, because the individual assay conditions were not fully optimized for quantitative affinity determination, the resulting *K*_D_ values should be regarded as method-dependent apparent affinities rather than definitive measures of intrinsic ligand affinity. A ligand-depletion-corrected analysis of the size-exclusion data and a matched-condition comparison of the different assay formats were beyond the scope of the present study and would be required for a rigorous quantitative comparison. Despite this limitation, the differences in the binding affinities of [^18^F]AG-120 determined using recombinant IDH enzymes or isolated cytosolic fractions appear insufficient to explain the previously observed different relationship between IDH1 mutation status and [^18^F]AG-120 accumulation. Other factors, such as tumor perfusion or the tumor microenvironment, may contribute to this discrepancy but were not investigated in the present study.

All binding measurements were performed in the presence of 10 mM Mg^2+^ but without addition of NADP^+^, NADPH, isocitrate or 2-OG. This simplified experimental approach was selected to avoid additional equilibria associated with cofactor and substrate occupancy, although the inhibitory potential of allosteric mIDH inhibitors may depend on the conformational and cofactor state of the enzyme (Deng et al. 2015, Liu et al. 2021, Liu et al. 2023). To assess the influence of NADP+/NADPH or substrate occupancy on [^18^F]AG-120 affinity, dedicated radioligand binding studies under defined cofactor and substrate conditions would be required.

Although not all experimental conditions were fully optimized, the present study demonstrates the applicability of established biochemical and biophysical techniques to radioligand binding studies of soluble intracellular proteins. In contrast to membrane-associated targets, for which radioligand binding assays are well established, comparatively few assay formats have been described for soluble intracellular proteins. The observed method-dependent differences in binding parameters further emphasize the importance of assay conditions when characterizing radioligand binding to soluble targets.

## Conclusions

The present study provides evidence for specific binding of [^18^F]AG-120 to both IDH1^wt^ and IDH1^R132H^ using established biochemical and biophysical assay formats. Recombinant proteins and cytosolic fractions from engineered cells provide experimentally accessible systems representing complementary and orthogonal approaches to characterize radioligand binding to soluble intracellular proteins. Although binding was consistently detected, the variation in apparent *K*_D_ values highlights the influence of assay conditions on quantitative affinity estimates. However, the investigated methods provide experimentally accessible approaches for the early-stage evaluation of radiotracers targeting soluble intracellular proteins.

## Methods

**Cell culture.** Human U251 glioblastoma cells, stably transfected and overexpressing either wild-type IDH1 (IDH1wt-U251) or IDH1R132H (IDH1R132H-U251), were obtained from Jaqueline Kessler and Dirk Vordermark (Department of Radiotherapy, Martin Luther University Halle-Wittenberg, Halle/Saale, Germany). Cells were grown in selective RPMI 1640 medium, supplemented with 10% FCS, 100 U/ml penicillin, 100 µg/ml streptomycin and 5 µg/ml puromycin to maintain selection pressure and ensure stable propagation of the transfected cell lines.

**Preparation of IDH1-containing cytosolic fraction.** At approximately 90% confluency, cells cultured in 175 cm^2^ flasks were washed twice with PBS without Ca^2+^ and Mg^2+^, detached gently using a cell scraper to maintain membrane integrity, and collected by centrifugation (1,000 rpm, 3 min, room temperature). To prepare whole-cell lysates, the sedimented cells were resuspended in ice-cold hypotonic buffer (50 mM TRIS-HCl, pH 7.4 at 4 °C; 5 µl per 10^6^ cells), followed by storage at -25 °C for at least 24 h to disrupt cells by ice crystal formation. On the day of the experiment, the lysate was thawed on ice and centrifuged at 15.000 rpm (rcf = 20.000) for 25 min at 4 °C to pellet nuclei and membranes. The resulting cytosolic supernatant was collected and used for subsequent experiments

**Determination of protein concentration.** Protein concentrations were determined using a BCA assay kit (Pierce™, Thermo Fisher Scientific) according to the manufacturer’s instructions.

**Preparation of test compounds.** Test compounds were prepared as 10 mM stock solutions in DMSO and diluted in the respective assay buffers to the desired final concentrations. Corresponding DMSO-containing solutions served as vehicle controls.

**Thermal shift assay.** The cytosolic supernatant was diluted with buffer (20 mM Tris, 150 mM NaCl, 10 mM MgCl₂, pH 7.5) to a final protein concentration of approximately 0.5 mg/mL. Aliquots of 1.1 mL were incubated with either 2 µL DMSO (vehicle control) or 2 µL of a 10 mM AG-120 solution in DMSO in 1.5 mL Eppendorf tubes on a rocking platform at room temperature for 60 min. Subsequently, 100 µL aliquots of each sample were transferred to fresh 0.5 mL Eppendorf tubes and incubated at the indicated temperatures in a thermocycler (Eppendorf ThermoMixer C; Eppendorf SE, Hamburg, Germany) at 2,000 rpm for exactly 3 min. Following incubation, samples were immediately transferred onto ice and centrifuged at 15,000 rpm and 4 °C for 20 min. An aliquot of 80 µL of the supernatant was carefully transferred to a fresh tube and stored at − 25 °C until electrophoretic separation and subsequent Western blot analysis.

Band intensities in Western blots generated from thermal shift assay samples were quantified by densitometry using AIDA Image Analyzer software (Elysia-Raytest GmbH, Straubenhardt, Germany) and expressed as a percentage relative to the intensity measured at 40 °C. These values were plotted against temperature, and the resulting data were fitted to a four-parameter sigmoidal curve (GraphPad Prism, 11.0.2). The midpoint of the curves, representing the temperature at which 50% of the initial band intensity remained (T_agg_), were determined from the fit.

**Native PAGE and autoradiography.** Cytosolic fractions prepared from approximately 2 × 10^6^ cells were used for each experiment, with a final protein concentration of 0.4–0.6 mg/mL. To 90 µL of the respective cytosolic fraction or buffer (50 mM sodium phosphate, pH 7.0, 150 mM NaCl), 10 µL of a working solution of [^18^F]AG-120 were added, resulting in a final radioactivity concentration of approximately 0.2 MBq/mL and a final ligand concentration of approximately 1 nM. Samples were incubated in glass vials at ambient temperature with shaking (200 rpm) for 60 min. At the end of the incubation period, 100 µL of 2× Tris-Glycine native sample buffer, containing 0.02% bromophenol blue (#42528; SERVA Electrophoresis GmbH, Heidelberg, Germany) were added. Proteins, together with a native molecular weight marker (NativeMark™, #57030; Invitrogen/Thermo Fisher Scientific, Waltham, MA, USA), were separated on 4–12% Tris-Glycine polyacrylamide gels (#43273; SERVA Electrophoresis GmbH) using Tris-Glycine Native Electrophoresis Buffer (#42530.01; SERVA Electrophoresis GmbH) at 200 V for 1 h.

Following electrophoresis, images of the gels were acquired to document the positions of the sample wells and the dye front. The gels were then exposed to phosphor imaging plates for 2 h together with radioactive reference markers indicating the positions of the sample wells and the dye front. The reference markers consisted of rectangular strips of filter paper spotted with diluted [^18^F]AG-120 solution in a predefined pattern. Subsequently, the phosphor imaging plates were scanned using a phosphor imager (HD-CR 35 NDT; DÜRR NDT GmbH & Co. KG, Bietigheim-Bissingen, Germany), and the gels were further processed for Western blot analysis (details see below). Finally, migration distances between the sample wells and the immunoreactive bands detected on the PVDF membranes were determined and compared with the positions of the radioactive signals obtained by phosphor imaging.

**SDS-PAGE and Western Blotting.** Samples were diluted in non-reducing Tris-Glycine/LDS sample buffer (42525; SERVA Electrophoresis GmbH, Heidelberg, Germany) and heated to 70 °C for thermal shift assay samples or to 95 °C for 5 min for all other samples prior to electrophoresis. Proteins were separated on 4–12% Tris-Glycine polyacrylamide gels (#43273; SERVA Electrophoresis GmbH) at 120 V for 1 h.

For Western blot analysis, proteins were transferred semi-dry onto PVDF membranes using continuous Bjerrum and Schafer-Nielsen transfer buffer (48 mM Tris, 39 mM glycine, 0.0375% SDS, and 20% methanol) at a constant current of 120 mA for 90 min. Successful protein transfer was verified by Ponceau S staining of the membrane. Following transfer, gels were stained with Coomassie Brilliant Blue (Quick Coomassie^®^ Stain, #35081.01; SERVA Electrophoresis GmbH) to assess residual protein content. Membranes were incubated with the respective primary antibody diluted 1:1000 (anti-IDH1: #DNA-DIA-W09, BIOZOL GmbH, Hamburg, Germany; anti-IDH1R132H: #DNA-DIA-H09, BIOZOL GmbH, Hamburg, Germany; anti-GAPDH: #GTX89409, GeneTex, Inc., CA, USA), followed by incubation with a fluorescence-labelled secondary antibody diluted 1:1000 (goat anti-rat, goat anti-mouse, donkey anti-goat; all Alexa Fluor^TM^647, Invitrogen/Thermo Fisher Scientific, Waltham, MA, USA). Immunoreactive bands were visualized by fluorescence detection using an Amersham Typhoon biomolecular imager (Cytiva, Marlborough, MA, USA). Band intensities in Western blots generated from thermal shift assay samples were quantified by densitometry using AIDA Image Analyzer software (Elysia-Raytest GmbH, Straubenhardt, Germany). The resulting intensity values were used to generate thermal denaturation curves and determine apparent aggregation temperatures.

**Size-exclusion chromatography. **The cytosolic fractions of approximately 1 × 10^8^ cells were used for a single experiment, comprising 6 samples of 120 µL each. Aliquots of the cytosolic fraction were distributed into separate glass vials and incubated with [^18^F]AG-120 (final concentration approximately 1–3 nM; ~ 1 MBq/mL) in the presence of either buffer (total binding), BAY1436032 (830 nM; final concentration; nonspecific binding) or varying concentrations of AG-120 (830 nM, 83 nM, 8.3 nM, 0.83 nM; final concentrations) for 120 min at room temperature. The incubation was terminated by pipetting 100 µL of each sample (containing ~ 250 µg protein) onto the resin bed of a manually prepared size-exclusion column of G25 fine resin (~ 4.5 mL resin/5 mL syringe), equilibrated with elution buffer containing 0.05 M Na-phosphate, pH 7.0, and 150 mM NaCl. Elution was performed by stepwise application of 200 µL of the elution buffer and eluted fractions were collected up to fraction 15 (total elution volume: 2.9 mL).

The activity in the eluted fractions was measured in a gamma-counter. Protein concentrations of the original cytosolic preparation and of eluted fractions with relevant activity levels were determined by BCA assay. Activity was plotted against fraction number or elution volume, and the area under the curve (AUC) was estimated by non-linear regression (GraphPad Prism 11.0.2). Specific AUC was calculated by subtracting nonspecific from total AUC, and percent of specific binding was determined for each concentration of the test compound. Percentage of specific binding was plotted against log [compound concentration] and nonlinear regression was used to generate sigmoidal dose-response curves. The IC50 and apparent dissociation constant (app *K*_D_) values were determined by fitting the data to the specific binding with variable Hill slope model using GraphPad Prism 11.0.2.

**Filtration assay. **The cytosolic fractions of approximately 2 × 10^8^ cells were used for a single filtration experiment, comprising 48 samples of 1 mL each. The cytosolic fractions were thawed and resuspended in the required volumes of assay buffer (20 mM Tris, 150 mM NaCl, 10 mM MgCl₂, 0.00045% Tween 20, 0.2 mM DTT, pH 7.5). Aliquots were distributed into separate glass vials and incubated with [^18^F]AG-120 (final concentration ~ 3 nM; ~ 1 MBq/mL) in the presence of either buffer (total binding), BAY1436032 (1 µM, final concentration; nonspecific binding) or varying concentrations of AG-120 (1 µM, 100 nM, 10 nM, 1 nM, 0.1 nM; final concentrations) for 120 min at room temperature. The incubation was terminated by filtration through glass fiber filters (GF 51, Hahnemühle FineArt GmbH, Dassel, Germany), which had been pre-soaked in freshly prepared 0.3% polyethyleneimine for 120 min at room temperature, using a 48-well cell harvester (Brandel Inc., Gaithersburg, MD, USA). Filters were subsequently washed three times with cold Tris-HCl buffer (50 mM, pH 7.4, 4 °C). Individual filter spots were then removed, and the filter-bound radioactivity was measured using an automated gamma-counter (2480 Wizard; Revvity, Hamburg, Germany) along aliquots from the working solution of [^18^F]AG-120.

Specific binding was calculated by subtracting nonspecific binding from total binding, and percent of specific binding was determined for each concentration of the test compound. Percentage of specific binding was plotted against log [compound concentration] and nonlinear regression was used to generate sigmoidal dose-response curves. The IC50 and apparent dissociation constant (app *K*_D_) values were determined by fitting the data to the specific binding with variable Hill slope model using GraphPad Prism 11.0.2.

**Binding Affinity Determination Using Purified His-Tagged Proteins**. Purified His-tagged proteins (IDH1-N-His, Abcam, ab113858; IDH1R132H-C-6His, Active Motif, 31614) were immobilized onto Ni-NTA-coated 96-well plates (Ni-NTA HisSorb Plate, Qiagen, 35061) by incubation with ~ 500 ng protein per well in PBS for 12 h at 4 °C. Unbound protein was removed by washing three times with PBS. Binding experiments were performed using [^18^F]AG-120 at a fixed concentration of about 3 nM (~ 1 MBq/mL) alone (total binding) or supplemented with AG-120 at different concentrations ranging from 1 to 1000 nM in IDH assay buffer (20 mM Tris, 150 mM NaCl, 10 mM MgCl₂, 0.000045% Tween 20, 0.2 mM DTT, pH 7.5). Nonspecific binding was determined in the presence of 1 µM BAY1436032. Incubation (120 min, ambient temperature, orbital shaker) was terminated by aspiration of the incubation solution followed by washing three times with PBS. Afterwards, the well plate was exposed to a phosphor imaging plate overnight. Subsequently, the plate was scanned using a phosphor imager (HD-CR 35 NDT; DÜRR NDT GmbH & Co. KG, Bietigheim-Bissingen, Germany). Spot intensities obtained from the 96-well plate autoradiography were quantified by densitometry using AIDA Image Analyzer software (Elysia-Raytest GmbH, Straubenhardt, Germany). Specific binding was calculated by subtracting nonspecific binding from total binding, and percent of specific binding was determined for each concentration of the test compound. Percentage of specific binding was plotted against log [compound concentration] and nonlinear regression was used to generate sigmoidal dose-response curves. The IC_50_ and apparent dissociation constant (app *K*_D_) values were determined by fitting the data to the specific binding with variable Hill slope model using GraphPad Prism 11.0.2

**Radiotracer production.** [^18^F]**AG-120** was produced on the TRACERlab FX2 N radiosynthesis module (GE Healthcare, USA) according to the procedure described earlier (Lai et al. 2024) with a high radiochemical purity of ≥ 99%, a radiochemical yield of 4.5 ± 1.0% (*n* = 11, EOB) and molar activities in the range of 191–678 GBq/µmol (*n* = 11, EOS) with starting activities of 20–30 GBq. 

## Supplementary Information

Below is the link to the electronic supplementary material.

Supplementary Material 1

## Data Availability

The datasets used and/or analyzed during the current study are available from the corresponding author on reasonable request.

## Abbreviations

2D: 2-Dimensional cell culture
2-HG: D-2-hydroxyglutarate 2-HG
2-OG: 2-Oxoglutarate 2-OG
IDH1: Isocitrate dehydrogenase 1
mIDH1: mutant IDH1 mIDH1
Ni-NTA: Nickel-nitrilotriacetic acid
PAGE: Polyacrylamide gel electrophoresis
PEI: Polyethylenimine
rcf: relative centrifugal force
