# Supplementary material for: Methods for assessing binding of a small-molecule radioligand to soluble enzyme isocitrate dehydrogenase 1 variants
Source: EJNMMI Radiopharm Chem. 2026 Sep 22;11:70. doi: 10.1186/s41181-026-00502-7 (PMC13598053; doi:10.1186/s41181-026-00502-7)
Supplement: Supplementary file 1 [file 41181_2026_502_MOESM1_ESM.docx]

**EJNMMI Radiopharmacy and Chemistry - Short Communications**

**Methods for Assessing Binding of a Small-Molecule Radioligand to Soluble Enzyme Isocitrate Dehydrogenase 1 Variants**

**Supplemental Information**

Winnie Deuther Conrad^1#^, Thu Hang Lai^2^, Andreas Maurer^1, 3^, Barbara Wenzel^1^

^1^ Department of Experimental Neurooncological Radiopharmacy, Institute of Radiopharmaceutical Cancer Research, Research Site Leipzig, Helmholtz-Zentrum Dresden-Rossendorf (HZDR), 04318 Leipzig, Germany

^2^ Department of Research and Development, ROTOP Pharmaka GmbH, 01328 Dresden, Germany

^3^ Institute for Drug Discovery, Leipzig University, 04103 Leipzig, Deutschland

**Figure S1.** Confirmation of the presence of the targeted protein in the size-exclusion chromatography fractions showing high concentration of the radioligand (representative experiment).

**Figure S2.** Control size-exclusion chromatography experiment comparing the elution profiles of [^18^F]AG-120 in the absence and presence of the cytosolic fraction derived from IDH1^R132Ht^-U251 cells (representative experiment).

**Figure S3.** Saturation curve analysis of the data obtained by size-exclusion chromatography of the cytosolic fraction derived from IDH1^wt^-U251 cells (representative experiment).

**Figure S4.** Saturation curve analysis of the data obtained by size-exclusion chromatography of the cytosolic fraction derived from IDH1^R132H^-U251 cells (representative experiment).

**Figure S5.** Control glass fibre filtration binding assay including parental U251 cytosol as IDH1-specificity control (representative experiment).

**Figure S1.** Size-exclusion chromatography (SEC) of the cytosolic fraction derived from IDH1^wt^-U251 cells. Confirmation of the presence of the targeted protein in the fractions showing high concentration of the radioligand. (A) Activity vs. eluted fraction, (B) Protein concentration of the activity-containing fractions, (C) western blot of electrophoretically separated activity-containing fractions (anti-IDH1: #DNA-DIA-W09, anti-GAPDH: #GTX89409). Representative experiment (2025-08-12).

**Figure S2.** Size-exclusion chromatography (SEC) of the cytosolic fraction derived from IDH1^R132Ht^-U251 cells. Control experiment comparing the elution profiles of [^18^F]AG-120 in the absence (open circles, [^18^F]AG-120 only) and presence of protein (filled circles, IDH1R132H + [^18^F]AG-120). In the absence of protein, the radioligand did not co-elute with the protein, and most of the radioactivity was still associated with the column at the end of the elution protocol. Representative experiment (2024-11-26).

**Figure S3.** Size-exclusion chromatography (SEC) of the cytosolic fraction derived from IDH1^wt^-U251 cells. Saturation curve analysis based on SEC data obtained from samples of the homologous competition assay. Representative experiment (2025-08-12). The apparent K_D_ value determined for IDH1^wt^ was 617 nM.

**Figure S4.** Size-exclusion chromatography (SEC) of the cytosolic fraction derived from IDH1^R132H^-U251 cells. Saturation curve analysis based on SEC data obtained from samples of the homologous competition assay. Representative experiment (2024-11-26). The apparent K_D_ value determined for IDH1^R132H^ was 42.9 nM.

**Figure S5.** Glass fiber filtration binding assay including parental U251 cytosol as IDH1-specificity control. Representative experiment (2026-03-09).
